# Recent advance in the discovery of tyrosinase inhibitors from natural sources via separation methods

**DOI:** 10.1080/14756366.2021.1983559

**Published:** 2021-09-27

**Authors:** Xiao-wei Zhang, Guang-li Bian, Pei-ying Kang, Xin-jie Cheng, Kai Yan, Yong-li Liu, Yan-xia Gao, De-qiang Li

**Affiliations:** a Department of Neurological Surgery, the Second Hospital of Hebei Medical University, Shijiazhuang, China; b Department of Pharmacy, the Second Hospital of Hebei Medical University, Shijiazhuang, China; c Department of Clinical Laboratory, the Second Hospital of Hebei Medical University, Shijiazhuang, China; d Institute for Drug Control of Hebei Province, Shijiazhuang, China

**Keywords:** Tyrosinase inhibitors, natural products, rapid screening, separation methods

## Abstract

Tyrosinase (TYR) inhibitors are in great demand in the food, cosmetic and medical industrials due to their important roles. Therefore, the discovery of high-quality TYR inhibitors is always pursued. Natural products as one of the most important sources of bioactive compounds discovery have been increasingly used for TYR inhibitors screening. However, due to their complex compositions, it is still a great challenge to rapid screening and identification of biologically active components from them. In recent years, with the help of separation technologies and the affinity and intrinsic activity of target enzymes, two advanced approaches including affinity screening and inhibition profiling showed great promises for a successful screening of bioactive compounds from natural sources. This review summarises the recent progress of separation-based methods for TYR inhibitors screening, with an emphasis on the principle, application, advantage, and drawback of each method along with perspectives in the future development of these screening techniques and screened hit compounds.

## 1. Introduction

Tyrosinase (TYR), also known as polyphenol oxidase, is a copper-containing metal enzyme that is widely found in different organisms and plays an important role in melanogenesis and enzymatic browning^1^^,^^2^. Therefore, its inhibitors are extensively used in cosmetics and medicinal industries as depigmentation agents and also in food and agriculture industries as antibrowning compounds^3^. At present, there have been countless studies on TYR inhibitors derived from natural or synthetic sources, most of which were only tested using TYR isolated from the mushroom due to its being readily commercially available. However, the TYRs derived from different sources such as mushroom tyrosinase (mTYR) and recombinant human tyrosinase (hTYR) were shown to have different specificities, causes different reactions of the screened substances on them^4^^,^^5^. Some TYR inhibitors that have been screened based on the mTYR have limited clinical efficacy, which encourages researchers to commit themselves to look for higher quality TYR inhibitors^6^.

Natural products have been recognised as one of the most excellent pools of lead compounds discovery because of their untapped chemical diversity and biological relevance^7^^,^^8^. However, the components of natural products are very complex, making the rapid discovery of the specific bioactive compounds from them a challenging task. Traditional screening strategies of extraction-isolation-bioassay and bioassay-guided fractionation are time-consuming and labour-intensive for active ingredients discovery^9^^,^^10^. Fortunately, with the continuous development of technology in recent years, some high-throughput screening methods such as ultrafiltration affinity screening, immobilised enzyme affinity, and HPTLC-autography have been established for Tyr inhibitors screening from natural sources (Table 1). By combining these modern separation techniques with the affinity or intrinsic activity of enzymes, affinity screening and inhibition profiling have become the two recent screening strategies. However, most of the current studies only focussed on the introduction of a certain method, the comparisons and applications of these strategies for TYR inhibitors screening have not been summarised yet comprehensively. Considering screening tools may cause confusion, with difficulty in selecting suitable ones due to their considerable diversity, merits and limitations, therefore, this article reviews the screening methods of TYR inhibitors developed in recent years via separation strategies, mainly introducing their principles, applications, advantages and drawbacks as well as the development trends in future.

**Table 1.** Applications of modern separation strategies for rapid screening of natural TYR inhibitors.

Methods	Source	Source of TYR	Active ingredients	References
UF-HPLC/MS	*Pueraria lobata Ohwi*	Unknown	3′-Hydroxypuerarin^1#^, Puerarin^2^, Puerarin-6''-O-xyloside^33^, Daidzin^34^, Genistin^35^, 6''-O-acetyldaidzin^36^, Daidzein^37^	^11^
	Semen Oroxyli	Mushroom	Oroxin B^3^, Kaempferide-3-O-β-D-gentiobioside^4^, Chrysin-7-O-β-D-gentiobioside^5^, Oroxin A^6^, Chrysin-7-O-β- D-glucuronid^7^, Baicalein^8^, Chrysin^9^	^12^
	*Xanthii fructus*	Mushroom	Protocatechuic acqid^82^, 3,5-Di-O-caffeoylquinic acid^57^, 1,5-Di-O-caffeoyluinic acid^58^, Chlorogenic acid^59^	^13^
	Mulberry leaves	Mushroom	Quercetin-3-O-(6-O-malonyl)-β-D glucopyranoside^14^, Neochlorogenic acid, Kaempferol-3-O-(6-O-malonyl)-β-D-glucopyranoside^15^, Chlorogenic acid^59^, Cryptochlorogenic acid^60^, 3,4-Dicaffeorylquinic acid^61^, 3,5-Dicaffeorylquinic acid^62^, 4,5-Dicaffeorylquinic acid^63^, Rutin^16^, Isoquercitrin^17^, Astragalin^18^, Kaempferol-3-O-α-L-rhamnopyranosyl-(1-6)-β-D-glucopyranoside^19^	^14^
	*Puerariae lobatae* radix	Unknown	Puerarin^2^, Mirificin^38^, Daidzin^34^, Genistinc^39^	^15^
UF-HSCCC-HPLC	*Otholobium pubescens* (Poir.) J.W. Grimes	Mushroom	Daidzin^34^, Isoorientin^10^, Vitexin^11^, Isovitexin^12^, Isoorientin 3′-methyl ether^13^, Daidzein^37^, Genistein, 3-(5-Hydroxybenzofuran-6-yl) propanoic acid^94^	^16^
	*Gastrodia elata*	Mushroom	12-15 Heneicosadienoic acid, Octadecanoic acid, 2-Hydroxylpropyl esters^119^, 3-(4-tert-butylphenyl)-2-Methylpropanal^120^, 4,4'-Dihydroxybenzylsulfid^121^, Octadecanoic acid, 2-Hydroxy-1-(hydroxymethyl)ethyl ester^122^, 9-Octadecenamide, (trans-)^95^, 9-Octadecenamide, (cis-)^96^, 4,4'-Dihydroxydiphenyl ether^64^, 4-(1-(4-hydroxyphenyl)phenyl)Phenol^65^, 2,3-Dihydroxypropyl ester^117^, 4,4'-Dimethoxydiphenylmethane^118^, 4,4’-Methylenediphenol^66^, 2,4-Bis(4-hydroxybenzyl) phenol^67^,4-Hydroxybenzyl methyl ether^68^	^17^
	Mango leaves	Mushroom	Gallic acid^78^, Iriflophenone 3-C-glucoside^97^, Mangiferin^46^, Protocatechuic acid^82^, Iriflophenone 3-C-(2'-O-galloyl)-glucoside^98^, 6'-O-Galloyl-mangiferin^47^, Maclurin 3-C-(2'-O- p-hydroxybenzoyl)-glucoside^99^, Iriflophenone 3-C-(2',6'- di-O-galloyl)-glucoside^100^, Hyperoside^20^, Isoquercitrin^17^, Ethyl gallate^79^, Iriflophenone 3-C-(2'-O- p-hydroxybenzoyl)-glucoside^101^, Quercetin-3-O-xyloside^21^, 3-O-Galloyl shikimic acid^75^, 3-O-Galloyl quinic acid^69^, Maclurin 3-C-(2'-O-galloyl)-glucoside^102^, 3,5-Di-O-galloyl quinic acid^70^, 5-O-Digalloyl quinic acid^71^, Digallic acid^80^, Isomangiferin^48^, 1,4,6-Tri-O-galloyl glucoside^72^, Maclurin 3-C-(2',3'- di-O-galloyl)-glucoside, 1,3-Digalleoyl acetone^113^, 1,3,4,6-Tetra-O-galloyl glucoside^73^, 6'-O-(p-Hydroxybenzoyl) mangiferin^49^, Epicatechin gallate^76^, 1,2,3,4,6-Penta-O-galloyl glucoside^74^, Iriflophenone 3-C-(2',3',6'- tri-O-galloyl)-glucoside^104^, Luteolin-7-O-glucoside^22^, Quercetin-3-O-arapyranoside^23^, Quercetin-3-O-arafuranoside^24^, Kaempferol-3-O-glucoside^25^, Quercetin-3-O-rhamnoside^26^, Ethyl 2,4-dihydroxy-3-galloyl Oxybenzoate, Ethyl digallate^81^, 7-O-Methyl quercetin-3- O-rhamnoside^27^	^18^
	*Glycyrrhiza uralensis* root	Mushroom	Liquiritin apioside^50^, Neolicuroside^83^, Liquiritigenin^51^, Liquorice saponin G2^105^, Chrysoeriol^52^, Dihydrodaidzein^53^, Formononetin^55^, Glycyrrhisoflavanone^54^, Glycyrrhizic acid, Licoarylcoumarin^40^, Pratensein^56^	^19^
IMF-HPLC-MS	*Glycyrrhiza uralensis* root	Mushroom	Liquiritin apioside^50^, Neolicuroside^83^, Liquiritigenin^51^, Liquorice saponin G2^105^, Chrysoeriol^52^, Dihydrodaidzein^53^, Formononetin^55^, Glycyrrhisoflavanone^54^, Glycyrrhizic acid, Licoarylcoumarin^40^, Pratensein^56^	^19^
MSPE-HPLC-MS	San-Bai decoction	Mushroom	Gallic acid^78^, Albiforin^106^, Paeoniforin^107^, Liquiritin apioside^50^, Liquiritin^84^, Galloylpaeoniflorin, Ononin^85^, Isoliquiritigenin, Glycyrrhizic acid,Oxypaeoniflora, Benzoylpaeoniflorin, Benzoyloxypaeoniflorin, Mudanpioside C, Paeonolide, Apiopaeonoside	^20^
TYR-AHF-HPLC-MS	*Pueraria lobata*	Mushroom	Puerarin-4′-O-glucoside^41^, 3′-Hydroxy puerarin^42^, Puerarin^2^, Puerarin-6′’-O-xyloside^33^, 3′-Methoxy puerarin^42^, Puerarin apioside^43^,Daidzein^37^	^21^
Off-line 2 D HPLC-MS/MS	*Pueraria lobata*	Mushroom	Puerarin^2^, Puerarin-6′’-O-xyloside^33^, Mirificin^38^	^22^
EMMA-CE	9 kinds of TCMS	Mushroom	–*	^23^
	21 kinds of TCMS	Mushroom	–	^24^
IMER-CE	*Psoralea corylifolia*	Mushroom	–	^25^
	*Folium ginkgo*	Mushroom	–	
	19 kinds of natural extracts	Mushroom	–	^26^
(HP)TLC-autography	*Glycyrrhiza glabra*	Mushroom	Glabridin^44^	^27^
	*Ganoderma formosanum*	Mushroom	–	^28^
	sandalwood oil	Mushroom	α-Santalol^112^	^29^
	*Rhodiola sacra*	Mushroom	Naringenin^45^, 1-O-β-D-glucopyranosyl-4-allylbenzene	^30^
	*Calamagrostis viridiflavescens* (Poir.)	Mushroom	Kojic acid^114^	^31^
	*Cinnamomum cassia* essential oil	Mushroom	Cinnamaldehyde^115^	^32^
HPLC-MS-EIA	Lavender flowers	Mushroom	5-Hydroxymethyl-furfural^116^	^33^
SER	*Rheum officinale*	Unknown	Emodin^111^, Veraphenol-4′-O-β-D- glycoside^88^, 1, 2, 6-Trihydroxy-5-methoxy-7-(3-methylbut-2-enyl) xanthone, 7-Hydroxy-2-(2-hydroxy)propyl-5methyl-benzopyran-γ-one, 2-O-cinnamyl-galloyl glucose^89^, ω-Hydroxy aloe-emodin, l, 6-Digalloyl-2-cinnamon acyl glucose^90^, 1, 4, 5, 6-Tetrahydroxy-7, 8-bis(3-methylbut-2-enyl) xanthone^30^, Emodin methyl ether^109^	^34^
	*Salvia miltiorrhiza–Carthamus tinctorius*	Agaricus bisporus	Protocatechuic aldehyde^91^, Hydroxysafor yellow A^92^, Tanshinone IIA^108^	^35^
	*Morus alba* root	Mushroom	Mulberrofuran G^93^, Kuwanon G^31^, Kuwanon H^32^	^36^

*: Without active compounds; ^#^: the compound No. in Table 1S.

## 2. Screening methods based on target enzyme affinity

Modern pharmacological studies show that the first step of most drugs to act is to combine with their targets, which has become the theoretical cornerstone of affinity screening. Currently, affinity screening methods such as ultrafiltration (UF) and immobilisation combined with chromatographic techniques have been widely developed for inhibitors discovery, which has been recognised as one of the most convenient and effective methods for screening active ingredients from complex mixtures. Compared with traditional screening methods, these newly developed methods have higher selectivity and specificity, they not only save time and effort but also allow a small sample^37^.

### 2.1. Ultrafiltration affinity screening

**Figure 1.**
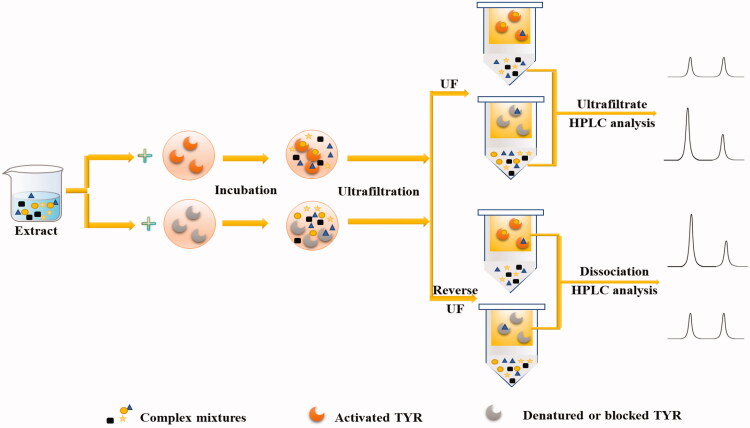
The procedures of two screening strategies are based on UF affinity.

As an affinity selection technology, UF can be used to screen active ingredients from complex mixtures quickly based on the molecular sieve principle of UF membranes^38^^,^^39^. The screening process is mainly to incubate the complex mixtures to be screened with the target enzyme in a mixing chamber containing a UF membrane. While the centrifugal ultrafiltration is performed, the conjugates are trapped and retained on the membrane and the unbound small molecules are eluted, the separation is completed in a flowing state^40^. The ultrafiltrate was collected and analysed by HPLC-MS. The fingerprint of the extract obtained initially is compared with that obtained from ultrafiltrate. A decrease in the peak areas on the fingerprint of ultrafiltrate indicates that the active compounds specially bind to the TYR. Or use the reverse UF method, that is, wash the retentate repeatedly to clean the remaining unbound small molecule compounds, and then release the active components from the target enzyme by changing the pH of the retentate solution or adding appropriate organic reagents, and then use mass spectrometry (MS) or other methods to identify active compounds. The procedures of these two screening strategies based on UF affinity were shown in Figure 1. This analytical process can maintain the natural conformation of the protein targets, and accurately reflect the physiological conditions for the interaction between the protein targets and small-molecule drugs.

At present, the UF-LC technology has been extensively used in the screening of TYR inhibitors. For example, the combination of UF-LC and molecular docking has been applied by Zhang et al. ^11^ and Yin et al. ^12^ to screen TYR inhibitors in *Pueraria lobata Ohwi* and Semen *Oroxyli*, respectively. 7 TYR inhibitors were screened in each experiment at last. Wang et al. ^13^ developed an Ultrafiltration-high performance liquid chromatography-diode array detector (UF-HPLC-DAD) method to screen and identify TYR inhibitors, and the false negatives were eliminated by reducing the background noise while false-positive results by using blocked TYR instead of enzymes as controls. Before the experiment, in order to obtain the best blocker, the competitive experiments were performed using variously known ligands. Four competitive TYR inhibitors were screened out successfully. Through the selection of optimal conditions including binding conditions, TYR concentration, and incubation time, Yang et al. ^14^ screened and identify 12 active compounds from mulberry leaves using the established UF-HPLC-DAD-MS method. UF-HPLC-QTOF-MS/MS strategy was applied by Liu et al. ^15^ to screen TYR inhibitors from *Puerariae lobatae* Radix, kojic acid was used to blocking the TYR-site to eliminate false positives. The activity results predicted by molecular docking are consistent with that obtained from *in vitro* activity. High-speed countercurrent chromatography (HSCCC), as a new type of high-efficiency chromatography technology, has been introduced to screen TYR inhibitors now. For example, through the combination of UF, HSCCC, and Pre-HPLC, Zuo et al. ^16^ screened 10 TYR inhibitors from *Otholobium pubescens* (Poir.) JW Grimes successfully. And the structure of 8 components was identified by nuclear magnetic resonance (NMR) or other methods. In order to verify the effectiveness of the established method, the *in vitro* enzyme inhibition activity and kinetics of the isolated compounds were determined finally. HSCCC was also employed to enrich the extract of *Gastrodia elata* by Wang et al. ^17^, UF-HPLC was used to screen TYR inhibitors subsequently, 17 ligands with high affinity were identified and separated, 14 of them were characterised by NMR and other methods successfully. In addition to the UF strategy, the efficient reverse UF is also widely used in the screening of TYR inhibitors. Shi et al. ^18^ proposed a new method that combined reverse UF-HPLC-QTOF-MS/MS with key ion filtering (KIF) strategies to screen TYR inhibitors in mango leaves. First, reverse UF-HPLC-QTOF-MS/MS was used to screen the TYR inhibitors in mango leaves, and then a key ion database was built through the analysis of the screened compounds, the KIF strategy was used to further explore potential TYR inhibitors in mango leaves. Finally, 36 TYR inhibitors were obtained successfully. Similarly, Liu et al. ^19^ developed a reverse UF-HPLC method and successfully applied it to the screening of TYR inhibitors in *Glycyrrhiza uralensis* root.

All these previous studies demonstrate that UF coupled with LC-MS can screen and identify active compounds quickly and accurately from a complex mixture. Compared with traditional methods, this strategy displays more advantages such as easy operation, time-saving, and low labour intensity^41^. However, there are still some drawbacks to this method. One of the most significant limitations is the false positive phenomenon resulting from the non-specific interactions between the target and the candidate screening substances. To reduce the false-positive results, repeated manual washing was commonly required to remove small molecules that are not bound to the active sites of enzyme^42^. Some components with weak interactions can also be removed. Therefore, this method is inappropriate for investigating the dissociation constant between bioactive components and targets. Alternatively, to eliminate this effect, inactive enzymes or competitive ligands can be used as parallel control samples, but the physical and chemical properties of inactive enzymes may have changed, making it difficult to compare with active enzymes. In addition, the non-specific interaction between components and UF membrane should not be ignored, especially when screening the ones with high lipophilicity. In the future, the competitive test of positive drugs and activity verification of bound ligands will be an option for reducing false results.

### 2.2. Immobilised target affinity screening

In recent years, significant progress was observed in the application of immobilised enzymes for the discovery of active compounds from complex mixtures^43^^,^^44^. The principle is to immobilise biologically active substances such as enzymes or cells on a specific carrier by physical, chemical, or affinity connection methods and incubate the samples with the carrier under suitable conditions so that the active ingredient can be combined with the biologically active substance, and then the bound ligand is dissociated by a certain method, at last, HPLC-MS/MS method is used to characterise the ligands to the identification of the active compounds screened out.

**Figure 2.**
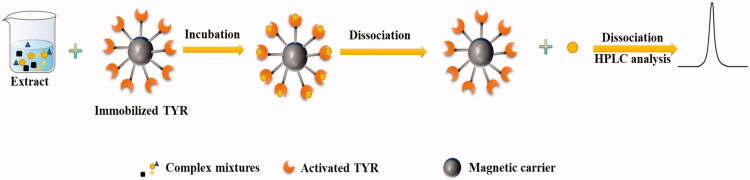
Schematic diagram of TYR inhibitor screening procedure based on TYR immobilised magnetic fishing.

**Figure 3.**
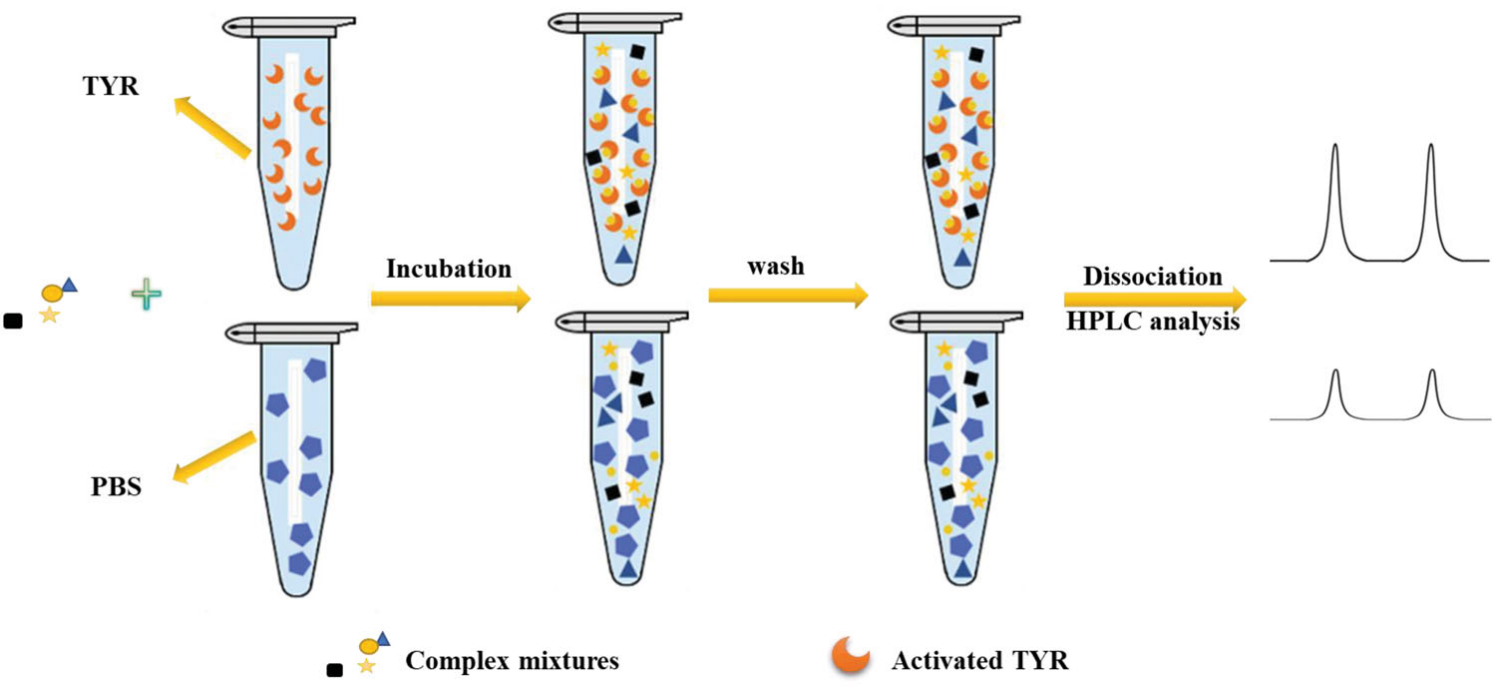
Schematic diagram of TYR inhibitor screening procedure based on TYR-AHF.

For example, Liu et al. ^19^ performed the TYR inhibitors ligand fishing from the roots of *Glycyrrhiza uralensis* based on TYR immobilised magnetic fishing (IMF) coupled with HPLC-DAD-MS/MS (Figure 2). The activity of immobilised TYR was obviously improved, retaining 76.3% after ten consecutive cycles, and over 95% when stored at 4 °C for about two months. Eleven TYR binders were successfully screened and characterised from *G. uralensis* root. Tao et al. ^20^ developed a method to analyse and identify TYR inhibitors from San-Bai decoction based on combined magnetic solid-phase extraction (MSPE) combined with HPLC-Q-TOF-MS/MS analysis. Before the experiment, paeoniflorin was used as a model compound to optimise the experimental conditions by response surface design, a total of fifteen TYR binders were identified and their inhibitions on TYR were finally verified by TYR inhibitory assay. Zhao et al. ^21^ established an adsorption hollow fibre (AHF) immobilised TYR method to screen TYR inhibitors (Figure 3), the reliability of which was firstly verified by kojic acid and ranitidine used as the positive and negative control, respectively. And, repeatability was carried out to confirm the accuracy of the method, seven potential TYR inhibitors were fished out from *P. lobata* successfully and their chemical structures were tentatively identified by LC-MS/MS, four of them were tested *in vitro* to verify their inhibitory activities, and molecular docking was performed at last.

Immobilised strategy can screen and identify TYR inhibitors from complex mixtures rapidly and its combination with LC-MS makes it possible to elaborate the structure of active compounds. Compared with free enzymes UF-HPLC assay, immobilised enzymes not only enhance the stability and durability but also prolong the survival time of the enzymes. Furthermore, the main advantage of immobilisation technology based on magnetic materials is the simple and rapid separation of materials and a mixed solution of samples, eliminating the tedious operation steps such as repeated centrifugation, which can achieve rapid separation and protect biomaterials. While, due to its advantages of simple operation, low cost and reusability, HF has gradually become a common immobilisation material. Despite these successes, limitations persist. As one kind of affinity screening method, immobilisation technology still has the problem of false positives, so how to eliminate non-specific binding is also a major challenge in the screening process. In addition, in the vast majority of cases, separation and analysis are independently done in off-line mode, with that screening is performed first and then the obtained potential active compounds are analysed by chromatography or MS. However, compared with online models, the offline mode could consume more sample and time during sample transfer and showed a much lower efficiency of screening and analysis. Therefore, in the future, efforts need to be made to develop online methods that could couple separation and analysis more efficiently.

### 2.3. Offline two-dimensional LC/MS affinity screening

**Figure 4.**
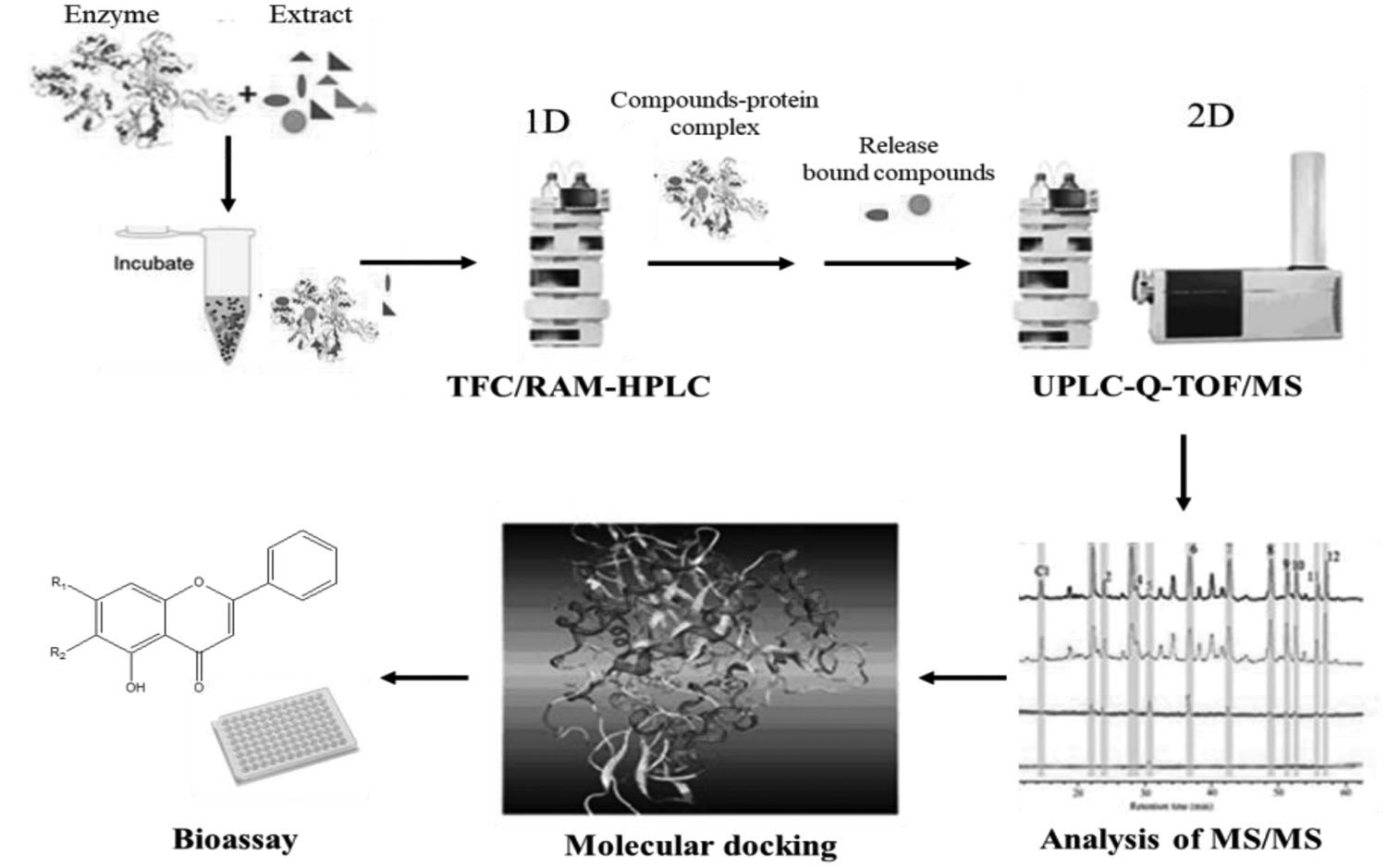
Schematic representation of the offline two-dimensional LC/MS for TYR inhibitors affinity screening.

The offline two-dimensional LC/MS technology (2 D LC/MS) includes two independent procedures: the separation of enzyme-ligand conjugates and unbound compounds and the dissociation, analysis, and identification of the bound ligands. Figure 4 showed the scheme of the solution-based free enzyme ligand screening by off 2 D-LC/MS. Firstly, the extract was incubated with an enzyme solution, and then separation of enzyme-ligand conjugates and unbound small molecules was performed by using turbo-flow chromatography (TFC) or restricted-access material (RAM). Based on the different sizes, small molecules can be retained, while the target-ligand conjugates can be quickly eluted. Thereby the rapid separation of conjugates and unbound small molecules was achieved, which is the first-dimensional chromatography. And then use a certain method to dissociate the conjugate and UPLC-MS/MS was used to analyse and identify the active compounds. This is second-dimensional chromatography.

This strategy was successfully carried out to discover TYR inhibitors from *P. lobata* by combing the TFC and LC-MS/MS analysis^22^. The ultra-fast separation of enzyme-ligand complex and unbound small molecules was achieved in less than 1 min in 1^st^ dimension. Subsequently, the ligands were dissociated from the enzyme-ligand complex with methanol precipitation, and then were injected into the 2^ed^ dimensional LC-MS/MS for ligands characterisation. As a result, three active ingredients were screened successfully and the inhibitory activities of selected TYR inhibitors were verified *in vitro*, which were consistent with those obtained by UF^11^ and HF immobilised enzyme affinity screening^21^, proving that this method was effective for bioactive compounds screening from a complex mixture.

Compared with the immobilised target LC/MS method, this method is a simple and convenient method with good reproducibility. Because enzyme solution is directly used instead of immobilised enzyme to incubate with extract, there is no need to choose a suitable carrier and prepare the immobilised enzyme, and the activity and stability of the enzyme after immobilisation do not need to be considered. While compared with the UF affinity method, the complicated repeated cleaning process can be avoided. However, in the process of achieving the separation of conjugates and small molecules, it is necessary to select a suitable chromatographic column which should be able to retain the unbound components, avoiding the false-positive results caused by its co-elution with the conjugates. At present, this newly developed method is not widely used. However, in terms of its advantages of fast, simple and automatic separation of enzyme-ligand complex and small molecules, the off-line 2 D LC-MS technology will have a good promise for screening bioactive compounds from complex mixtures.

## 3. Screening method based on the intrinsic activity of the target enzyme

Enzymes have biological activity and can catalyse substrates to produce products, so the activity of enzymes can be evaluated based on the changes in the amount of substrate or product. Combining this characteristic of enzymes with chromatographic technology can screen active ingredients in complex mixtures quickly and accurately.

### 3.1. Online CE-based methods

The CE-based screening methods combine the enzyme reaction and the separation of capillary electrophoresis (CE) for evaluating the bioactivity of drug candidates. Now the methods commonly used for screening mainly include electrophoretically mediated microanalysis (EMMA) and immobilised enzyme microreactor (IMER) ^45^. The EMMA screening method is realised by using the different mobility of enzyme and substrate in the capillary, which process is to add enzyme and substrate in the capillary by sequence, then apply voltage. Due to the difference in migration rate, the enzyme can be mixed with the substrate to react, and high voltage is applied to separate them at last, the enzyme activity is determined according to the change of peak area of the product. This EMMA method was first proposed by Bao et al. ^46^ and is now widely used in the screening of various enzyme inhibitors, more information about EMMA can be found in several previous reviews^47–49^. For TYR inhibitors screening, Tang et al. ^23^ developed an EMMA method by integrating the techniques of sandwich mode injection, partial filling, and rapid polarity switching, and by using it, on-line enzyme reaction and the separation of substrate and product were well carried out after optimising the conditions of background electrolyte, mixing voltage, and the incubation time. The inhibitors were directly identified from the reduced peak area of the product compared to those obtained without any inhibitor. As a result, one of nine standard natural compounds, chlorogenic acid, showed inhibitory activity on TYR. Zhang et al. ^24^ also established an EMMA method using longitudinal diffusion as the mixing technique, the inhibitory activities of 21 TCMs on TYR were determined.

On the other hand, the principle of IMER method is basically the same as EMMA method except that the enzyme is immobilised on the capillary by means of adsorption, bonding, embedding, etc. When screening TYR inhibitors, the complex components are mixed with the substrate solution, and the inhibitory effect of the complex components on the enzyme is detected by comparing the peak area of the substrate without samples. Cheng et al. ^25^ immobilised TYR at the outlet of the capillary using glutaraldehyde as a cross-linking agent to form IMER of TYR. By adopting a short-end injection procedure, the product and substrate were effectively separated within 2 min. The immobilised TYR could remain 80% active for 30 days at 4 °C. The TYR inhibition rate of 15 standard natural compounds was measured, and the relationship between the screened TYR inhibitors and TYR was studied through molecular docking. Jiang et al. ^26^ developed an IMRE based on a layer-by-layer assembly for TYR inhibitor screening. TYR was immobilised on the surface of fused silica capillary via ionic binding technique with cationic polyelectrolyte hexadimethrine bromide (HDB). Then, HDB solution was injected again into the capillary to cover the immobilised enzyme by forming HDB–TRS–HDB sandwich-like structure. Using L-Tyrosine as a substrate, the product L-dopa was used for the screening of enzyme inhibitors based on the reduction of its peak area, and finally, the TYR inhibition rate of 19 kinds of traditional Chinese medicine extracts was determined.

As an efficient separation approach, CE possesses many advantages such as less sample consumption, fast analysis speed, high efficiency, easy to realise automation, miniaturisation, etc.^50^, which can be applied to the screening of multiple enzyme inhibitors due to its multiple separation modes and many types of detectors. The online CE method allows the biochemical assay integrated into the separation process of CE so that avoids the possible pollution caused by the outside world. Among them, the EMMA does not need to modify capillary. But it needs to sample enzyme in each assay, which increases experiment cost especially when enzymes are expensive. The complicated sampling process and mixing in the capillary also result in more measurement errors. As compared with EMMA, the enzymatic reaction inside an IMER avoids these disadvantages. In addition, the IMER not only improves the stability of the enzyme but also can be reused, reducing the consumption of the enzyme and the cost. However, it needs to take a lot of effort to explore how to fix the enzyme on the capillary by a suitable method which not only will not affect the activity of the enzyme but will also increase the frequency of use of IMER. Furthermore, neither EMMA nor IMER, the CE-based methods are only used to perform a preliminary evaluation of enzyme inhibitory activity of screened substances by determining the change of substrate or product, direct screening of single active TYR inhibitors from complex natural products has not been reported yet. In the near future, CE-based enzyme inhibitor screening requires more and more new materials for enzyme immobilisation, new detectors for enhancing the resolution of the products and substrates, ultra-high-throughput screening, more speed analytical techniques, and computer simulation.

### 3.2. (High-performance) thin-layer chromatography ((HP)TLC)-autography

Among the existing screening technology of active compounds, (HP)TLC-autography is particularly useful^51^. The target reaction solution was employed as the colour developer, and the active compounds screening was depend on the colour change of the spot. It can not only separate the mixed components but also carry out biological activity detection. The study using the method of TLC-autography to screen enzyme inhibitors can be traced back to 1964, Menn et al. ^52^ used plasma instead of acetylcholinesterase to screen enzyme inhibitors. Since then, many researchers have carried out in-depth research on this method and the thin-layer chromatography (TLC) bio-autography assay has become very popular in screening for active compounds that may affect enzymes. The main steps are as follows: firstly, drop the mixed components on the thin-layer plate, separate the components with a suitable spreading agent, and then spray appropriate concentration of enzyme solutions and substrates on the thin-layer plate, at last screen out the active ingredients according to the different colours of the spots. This method can not only judge whether the ingredients contain enzyme inhibitors, but also separate the complex mixtures and locate the biologically active ingredients. (HP)TLC-autography is an effective method to screen active compounds in complex mixtures^53^.

Wangthong et al. ^27^ developed a TLC post-development technique for the screening of active ingredients of TYR inhibitors in liquorice. Using L-Tyrosine as a substrate, the positive results were could be visualised directly as white spot(s) against a brownish-purple background. The developed method was validated by detecting the TYR inhibitory activity of some known inhibitors including kojic acid, glabridin, arbutin, and was successfully used to analysis the liquorice extract. Similarly, this method was applied to screen the TYR inhibitors from *Ganoderma formosanum* extracts^28^ and Sandalwood oil^29^, respectively. To improve the sensitivity of detection and reduce enzyme consumption, an improved TLC-autographic assay for the discovery of TYR inhibitors was developed with L-DOPA as substrate which has better water solubility than L-Tyrosine. Two TYR inhibitory compounds were isolated from *Rhodiola sacra* guided by this TLC bioautographic assay^30^. Commonly, by using the (HP)TLC-autography, TYR inhibitors can be clearly identified as white spots against a dark background in white light remission as well as in white light transmitted through the plate. However, false-positive phenomenon was reported by Taibon et al. ^54^, that some lipophilic substances in the investigated extracts appeared as white spots in white light remission but were black in white light transmission due to poor wettability of the corresponding spots. To eliminate false-positive results, Triton X-100 was added to the substrate solution and the plate was dried after incubation with a molecular sieve. A variety of plant extracts were screened, and a TYR inhibitor from *M. alba* was screened and identified by MS successfully at last. When performing the (HP)TLC-autographic assay, the enzymatic activity is very prone to decrease in the process of drying out of the enzyme solution on the silica surface. To overcome this problem, enzyme immobilisation by gel entrapment was verified to be one ideal approach to increase enzyme stability in autographic assays^55^. García et al. ^31^^,^^32^ developed an autographic assay to detect TYR inhibitors using gel entrapped enzyme, a homogeneous agar gel layer was formed on a normal/reverse phase TLC surface. The applicability of this method was tested by detecting different concentrations of kojic acid and natural product extracts spiked with it.

The (HP)TLC-autographic method combines chromatographic separation and biological activity determination, which can be used to screen TYR inhibitors from complex mixtures simply, rapidly and efficiently, especially suitable for screening comparison among multiple samples simultaneously on a thin-layer plate. The choice of stationary phase and mobile phase is more flexible so it is easy to operate. Besides, this method can save costs as less sample volume and enzymes are required. The solvent can be evaporated quickly so that the enzyme activity is not affected by the solvent, and it is the only chromatographic method that enables the presentation of the results as pictures. However, this method still has some drawbacks, such as poor separation effect and low stability of enzyme. (HP)TLC has weak separation ability, in most cases, the components might be overlapped, so it is difficult to determine whether the biologically active is from a single component or a mixture of different compounds. Therefore, (HP)TLC-autographic method is mostly used as a tool for preliminary screening of active drug candidates. On the other hand, some enzymes have poor stability, drying the enzyme solution on the surface of the thin-layer plate will reduce the enzyme activity and reduce the contrast of the measurement^56^. How to fix the enzyme solution on the TLC plate without affecting the activity of the enzyme poses a challenge to the (HP)TLC-autographic method. Anyway, as a convenient screening method, the (HP)TLC-autographic is undoubtedly a good alternative for multi-batch and multi-activity comparison^57^.

As one kind of effect-directed analysis (EDA), TLC bio-autography has the most extensive applications in recent years and has been reviewed comprehensively^58–61^. In the future, there will be two possible development directions in technology. On the one hand, to overcome the poor separation efficiency of TLC, 2 D HPTLC can be adopted to get a higher peak capacity. On the other hand, for a long time, the lack of hyphenation between TLC and MS always hindered the efficiency of this method. With the commercialisation of some interfaces and the development of *in situ* detection, the “online” extraction of analytes from the plate prior to electrospray ionisation ESI-MS analysis can be achieved, paving the way for rapid dereplication of bioactive compounds from natural products.

### 3.3. HPLC-MS coupled with post-column enzyme inhibition assay

**Figure 5.**
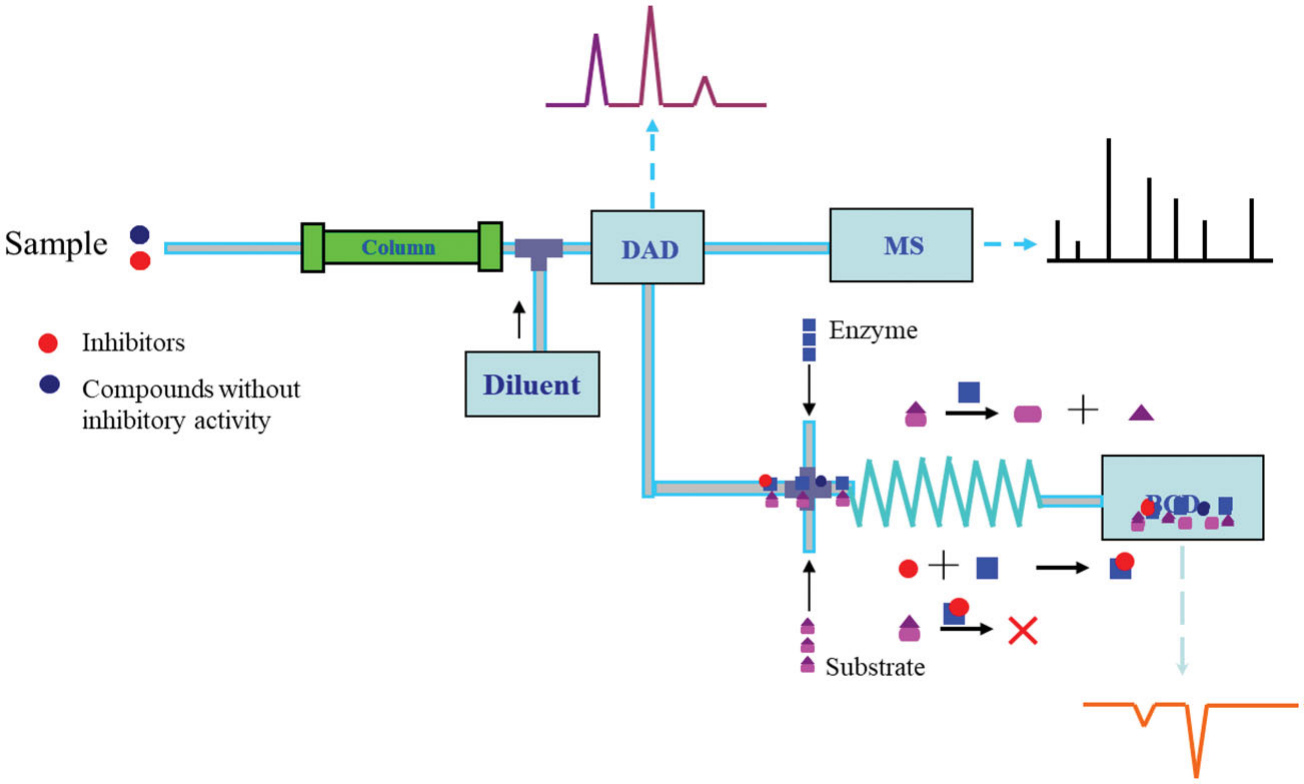
Schematic diagram of enzyme inhibitor screening procedure based on HPLC-MS coupled with post-column bioassay.

The HPLC-MS coupled with post-column enzyme inhibition assay (EIA) integrates chromatographic separation, compound identification and activity evaluation into one system, the biological activity information and chemical information of the compounds in the complex mixture can be obtained simultaneously through one injection. By using this approach, the complex mixtures are first separated by HPLC, then the compounds are eluted in sequence according to the polarity. The eluent from HPLC system is divided into two streams, one of which enters the MS directly to get a fingerprint, while another strand enters the reaction coil where enzyme and substrate are continuously pumped into. The inhibition profiling of the compounds is recorded by biochemical detection (BCD). If the ingredient has inhibitory activity on an enzyme, the amount of product will decrease, correspondingly, a negative peak will appear in the inhibition profiling (Figure 5**).** According to the fingerprints and post-column biochemical detection information, active ingredients can be screened out quickly, and the active ingredients can be qualitatively determined by MS^62^.

Luo et al. ^33^ established a new method for screening natural TYR inhibitors based on HPLC combined with the post-column EIA. In order to screen active compounds from complex mixtures, the conditions of the enzyme reaction including the amount of enzyme, the enzyme tolerance to organic solvents and reaction time were systematically optimised. The system was verified by the known potent TYR inhibitors, kojic acid, arbutin and hydroquinone, to determine the reliability of the developed method. Finally, the established method was successfully applied to screen bioactive ingredients in *lavender* flowers. The availability of three TYR inhibitors proves the effectiveness of this method for screening active ingredients in complex mixtures.

As an online method, this strategy is regarded as a more convenient and accurate manner in the screening of active components. Since the separation and analysis can be performed at the same time, the method can provide more intuitive results for the screening results, and real-time monitoring of bioactive information was realised. The method is simple in operation, fast in screening, and does not require purification and separation before activity detection. Compared with the offline experimental assays, this online strategy significantly saves analysis time, improves the accuracy of screening, and reduces the false positive rate^63^^,^^64^. Despite these extraordinary advantages, drawbacks persist. First, as a non-universal screening technique, each enzyme needs to find a suitable substrate for the reaction, facilitating the detection of inhibition profiling with high specificity, sensitivity, and stability and the concentration of the enzyme and the flow rate of the system need to be optimised. Second, the target enzyme needs to be continuously pumped into the reaction coil, which is costly^62^. Last but not the least, in order to avoid peak diffusion, the enzymatic reaction time should be strictly controlled, usually less than 2 min, which is prone to result from the false-negative results, cause some components with low abundant cannot show their inhibitory activity in such a short time. How to improve the applicability of this method to various enzymes is worthy of in-depth study.

In view of the above-mentioned limitations of HPLC-MS-EIA, there are some developed trends for it: (1) In order to minimalize the consumption of expensive enzymes/substrates, miniaturisation of online screening system is an alternative opinion, which can be carried out by the combination of the micro-scale HPLC elute and high sensitive detectors; (2) To avoid the strict time limits of the online post-column enzymatic reaction, an at-line nanofractionation analytics was proposed by Kool and co-workers^65–67^. In this approach, the post-column bioassay is a time-course functional assay based on a high-density well-plate, reducing the false-negative results due to the un-sufficient reaction time. (3) Natural products contain many compounds with a wide range of polarity, integration of different separation modes such as hydrophilic interaction chromatography (HILIC), RPLC, and GC with the at-line nanofractionation would be a direction for improving the identification quality of bioactive compounds. Compared to online assays generally requiring considerable method development and intricate implementation, the HPLC-based inhibition profiling in at-line or off-line mode are more versatile, with higher potential for wide application in drug discovery.

### 3.4. Screening method based on spectrum-effect relationship

The screening method based on spectrum-effect relationship (SER) is to link the biological activity of the sample with its chemical fingerprint^68^^,^^69^. The main step is to analyse the enzyme activity and fingerprints of many samples and establish a prediction model between the enzyme activity and the fingerprint through some data processing techniques such as partial least square (PLS) regression model or canonical correlation analysis. The enzyme activity of the compounds is predicted by the model, and then the selected active compounds are identified by HPLC-MS. Finally, the *in vitro* activity of the obtained active compound was verified. Exploring the SER may clarify overall therapeutic effects and the relationship between active constituents, which is widely applied in research into the basis for substance effectiveness^70^.

The screening of TYR inhibitors is more purposeful, is simple and reliable, and saves time and manpower. Liu et al. ^34^ established a screening method for TYR inhibitors based on the SER. The ethyl acetate extraction part of rhubarb was separated by HPLC, and the activity of the eluate collected at different time periods was measured, and the most active part was selected for UPLC-QTOF (quadrupole time of flight mass spectrometer) analysis to determine the composition. The analysed components were molecularly docked to predict their TYR inhibitory activity, and the predicted results were verified by *in vitro* activity determination. The results showed that the *in vitro* verification and prediction were completely consistent. This method can effectively screen TYR inhibitors. Wang et al. ^35^ employed the spectral effect to screen TYR inhibitors from *Salvia miltiorrhiza-safflowe.* c through canonical correlation analysis of the *in vitro* TYR inhibition rate and HPLC fingerprints of *Danshen-Safflower* mixed in different proportions to predict the chromatographic peaks that have a greater contribution to the inhibition of TYR, and then use HPLC-MS/MS to analyse and identify the compounds. A total of 13 active compounds were found, and *in vitro* activity measurements were performed on 5 of them to verify the predicted results. Finally, three TYR inhibitors were screened. Kang et al. ^36^ prepared 42 samples from 6 batches of root bark under 7 different extraction conditions for analysis, established a PLS regression model between fingerprint chromatography and biological activity, and predicted the active ingredients, screened three TYR inhibitors successfully.

This method shows some advantages such as reliability, time-saving capacity, and simple operation. Different from TLC- and HPLC-based EIA methods, the SER method can screen out the active ingredients with a lower content in TCM successfully. However, there are still many problems, such as the poor stability and repeatability of fingerprints of the complex mixture; Each data analysis method has its own advantages and disadvantages, which method needs to be further standardised; The design of efficacy experiment is not comprehensive enough to reflect the process of active substances entering the body. Being an interdisciplinary and cutting-edge science, the spectrum-effect relationship method is a technology integrating analytical chemistry, pharmacodynamics, and chemometrics. In the future, more professional talents need to be introduced to the development of related software engineering, exploring a wider world for the screening of effective compounds.

## 4. Perspectives of the combination of affinity and inhibition profiles

Though affinity screening and inhibition profiling both have the obvious advantage of the rapid discovery of TYR inhibitors directly from natural sources without any tedious purification procedures, the two approaches are established based on two different, and in some cases complementary, principles. Affinity screening is based on the binding affinity of molecules to the enzyme regardless of their inhibitory potential. However, the false-positive results are commonly caused in affinity screening by the non-specific affinity between compounds and enzymatic non-active sites or the solid support. While, for the mode of inhibition profiling, the inhibitory activity of compounds was measured according to the catalytic properties of the enzyme, some compounds like tannins have also been shown to result in false-positive results because of their non-specific interactions with the enzyme. Instead of the present strategies using either one, the combination of affinity screening and inhibition profiling would provide an opportunity to narrow down the hits with presumed bioactivity, minimising the risk of false-positive results, the proof-of-concept of which was demonstrated by α-glucosidase inhibitors screening from the crude ethyl acetate extract of *Ginkgo biloba*^71^. Undoubtedly, the combined approach provides a powerful tool for the screening of specifically target bioactive compounds with both specific affinity and activity.

## 5. Two important concerns for TYR inhibitors screening

Though the present review mainly focussed on the separation strategies for inhibitors screening, two important concerns for the TYR inhibitors discovery are worth to be noted. On one hand, mTYR rather than hTYR has been commonly used as the target for inhibitors screening due to cheap and commercially available. But, it was reported that inhibitors designed and evaluated against hTYR may have greater efficacy and lead to more effective treatments for pathological hyperpigmentation than those against mTYR^4^. Therefore, if the condition allows, it is better to use hTYR for inhibitors screening in the future. On the other hand, the previous report demonstrates that many plant-derived inhibitors such as polyphenols are in fact alternative substrates of mTYR and that might cause as well errors during the read-out with MS or UV absorption detection^72^. Since many polyphenols are found ubiquitously in natural extracts, a detailed understanding of the strengths and limitations of the used techniques is crucial.

## 6. Inhibitory activity and mechanisms of several screened compounds

**Figure 6.**
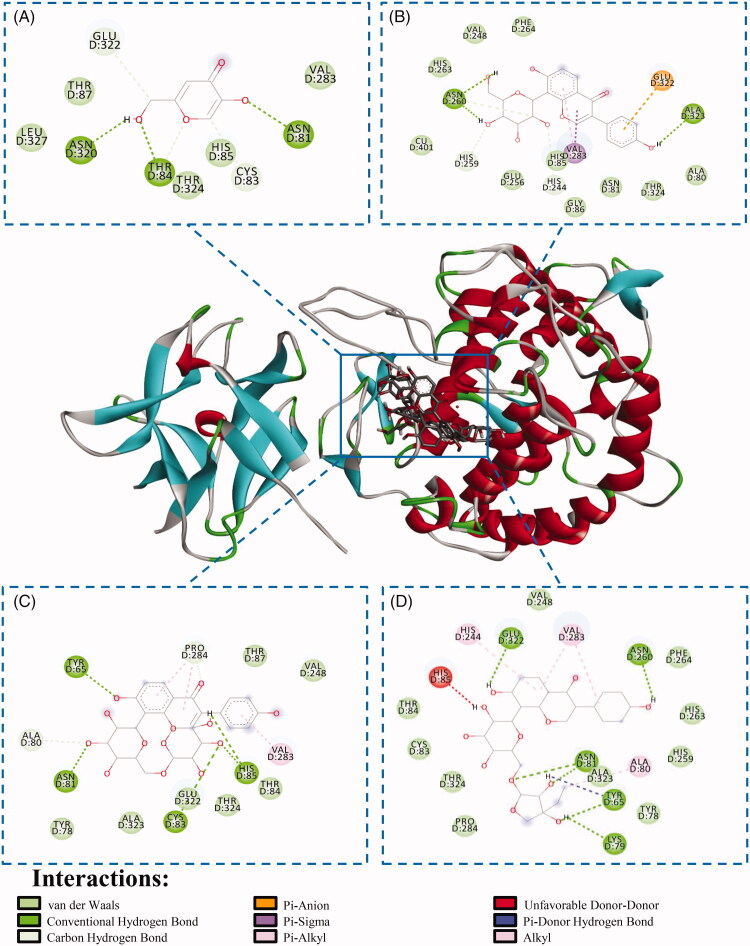
Molecular docking of kojic acid (A), puerarin (B), puerarin-6’’-O-xyloside (C), and puerarin apioside (D) with TYR (Copyrighted from Zhao et al. ^21^).

Using the above-described methods, a lot of TYR inhibitors have been found. However, the vast majority of these reports have focussed mainly on the determination of analytical parameters and analysis of samples with very little emphasis on the binding and the mechanism of inhibition. In order to further study the screened TYR inhibitors, enzyme inhibition assay and molecular docking had been usually carried out to explore their inhibitory mechanisms and binding characteristics. Among the TYR inhibitors derived from natural sources (Table 1S, please see the supplementary materials), those containing hydroxyl groups in their structures including flavonoids and polyphenols was turned to be effective for TYR inhibition probably due to chelating copper ions as well as binding critical residues affecting substrate access at the active site pocket, which generally displayed reversible and competitive inhibition types^73^^,^^74^ (Table 2). For example, to confirm the TYR inhibitory activity of hit compounds, four identified compounds from *P. lobata* extract was tested *in vitro*, and three of them, namely, puerarin, puerarin-6′’-O-xyloside and puerarin apioside were verified to have good TYR inhibitory activity with IC50 value of 478.5, 513.8, and 877.3 μM, respectively^21^. In addition, the molecular docking results indicated that these compounds could bind to the amino acid residues in TYR catalytic pocket (Figure 6**)** via multifarious interactions, including van der Waals, conventional hydrogen bond, carbon-hydrogen bond, Pi-Anion, Pi-Sigma and Pi-Alkyl forces. Similarly, by molecular docking, Zhang et al. ^11^ and Liu et al. ^15^ also discovered the importance of hydrogen bonding and π-cation interaction for binding. The more hydrogen bonds that can be formed, the stronger the biological or activity is. Puerarin-6′'-O-xyloside has more hydrogen interactions than puerarin because it has a xylose moiety, resulting in higher biological activity^11^. The docking results also showed that the four hydroxyl groups in puerarin formed four hydrogen bonds with TYR, and the ring B part of puerarin formed a π-cation interaction with the enzyme, which has a stronger inhibitory effect than the known TYR inhibitor kojic acid^11^. Liu et al. ^15^ Ranked the inhibitory activities of discovered hit compounds, which were as follows: puerarin > mirificin > Kojic acid > daidzin≈genistin, the molecular-docking analysis indicated that the addition of glycosyl reduced TYR inhibition due to steric hindrance and changes in polarity.

**Table 2.** The IC50, Ki, and inhibition mechanism of some screened compounds.

Compounds	IC50(mM)	Ki(mM)	Suppression mechanism	References
Puerarin^2^	1.23	/	/	^11^
	0.010			^15^
	0.479			^21^
	0.012			^22^
Oroxin A^6^	0.50			^12^
Baicalein^8^	0.29			
Protocatechuic acid^82^	/	9.28	Competitive inhibitor	^13^
3,5-Di-O-caffeoylquinic acid^57^		0.34		
1,5-Di-O-caffeoylquinic acid^58^		1.1		
Chlorogenic acid^59^		0.9	Mixed inhibitor	
Quercetin-3-O-(6-O-malonyl)-β-D glucopyranoside^14^	0.268	/	/	^14^
Kaempferol-3-O-(6-O-malonyl)-β-D-glucopyranoside^15^	0.104			
Mirificin^38^	0.013			^15^
Daidzin^34^	>500			
Genistinc^39^	>500			
Vitexin^11^	0.35			^16^
Isovitexin^12^	1.73			
Isoorientin^10^	7.67			
Isoorientin 3'-methyl ether^13^	8.61			
Daidzein^37^	1.58			
Genistein^/^	7.66			
3-(5-Hydroxybenzofuran-6-yl) propanoic acid^94^	1.33		Mixed-type inhibitor	
Gallic acid^78^	0.178		/	^20^
Albiforin^106^	0.100			
Paeoniforin^107^	0.102			
Liquiritin apioside^50^	0.089			
Liquiritin^84^	0.171			
Galloylpaeoniflorin^/^	0.036			
Ononin^85^	0.101			
Isoliquiritigenin^/^	0.185			
Glycyrrhizic acid^/^	0.059			
Oxypaeoniflora^/^	0.083			
Benzoylpaeoniflorin^/^	0.032			
Benzoyloxypaeoniflorin^/^	0.040			
Mudanpioside C^/^	0.083			
Paeonolide^/^	0.102			
Apiopaeonoside^/^	0.098			
Puerarin-6’’-O-xyloside^33^	0.514			^21^
Puerarin apioside^43^	0.877			
Emodin^111^	300mg/ml			^34^
Protocatechuic aldehyde^91^	0.455			^35^
Hydroxysafor yellow A^92^	0.498			
Tanshinone IIA^108^	1.214			
Mulberrofuran G^93^	0.018			^36^
Kuwanon G^31^	>0.2			
Kuwanon H^32^	0.010			

The importance of the chemical structure of the compound for biological activity has been confirmed by other studies. For example, dicaffeoylquinic acids showed more potent TYR inhibitory activities than the caffeoylquinic acids that may be attributed to the larger number of catechol moieties, which suggests that compounds with a catechol structure may have higher TYR inhibitory activity^14^. Tao et al. ^20^ found that the activity of paeoniflorin was slightly weaker than that of its derivatives, possibly due to the difference in its aromatic substituents. Furthermore, based on the spectrum-efficiency-structure-activity relationship, Liu et al. ^34^ screened TYR inhibitors in rhubarb. The biological activity of the compound was predicted through the results of molecular docking, and the TYR inhibition experiment was used to verify it. This study also demonstrated the important role of hydrogen bonding for binding. The 6-OH and 8-OH of emodin form hydrogen bonds with TYR, which has strong activity. Veratrol-4′-O-β-D-glucoside, containing meta-dihydroxyl structure, can form hydrogen bonds and complex interaction and also has strong biological activity. 2-O-cinnamyl-galloyl glucose also has a dual role of hydrogen bonding and complexation, which makes it have strong biological activity, but it may be weaker than the former two due to steric hindrance. The docking results of 1, 2, 6-trihydroxy-5-methoxy-7–(3-methylbut-2-enyl) xanthone with o-dihydroxy group shows that it is difficult for the o-dihydroxy group to form a hydrogen bond, so its activity is the weakest. In general, compounds that can form hydroxyl groups have strong biological activity but are also affected by steric hindrance and group polarity.

Undoubtedly, the enzyme inhibition assay and molecular docking will be conducive to understand the mechanism of the inhibitory effect of the hit compounds against TYR and provide the scientific basis for further development of them. However, up to date, the clinical trials with skin whitening agents or formulations hardly had been performed. A clinical study from Korea^75^ investigated the anti-melanogenesis activity for whitening products containing aerial part of *P. lobata* extract (APPL). After 4 weeks, subjects with 3% APPL treatment started to improve melanin content and visual evaluation on pigmentation and skin lightness. After 8 weeks of treatment, the Skin colours have been greatly improved. Safety evaluation proved that there was no adverse reaction on the skin upon treatment of products. Natural products have become the potential sources of cosmetics and medication to prevent skin hyperpigmentation. With the progress of screening technology, more and more lead compounds from them have been found, it is expected to carry out more clinical studies to confirm their effectiveness and safety.

## 7. Conclusion

In recent years, with the wide application of TYR inhibitors, searching for safe, specific, and effective TYR inhibitors has become a concern of researchers. Linking the powerful ability of separation technologies with enzyme characteristics, a series of screening methods have been developed, which have made great contributions to the development and application of TYR inhibitors.

This review summarises the current screening methods for TYR inhibitors developed based on chromatographic technology, introduces the basic principles of various screening methods, the advantages and drawbacks of various methods (Table 3), and their applications in the screening of complex systems such as TCM. In any case, the newly developed methods provide a powerful tool for screening active ingredients in complex compounds and greatly accelerate the speed of drug discovery compared with traditional methods. In the future, simpler, faster, and increasingly accurate modern bioanalytical techniques are expected to develop for the screening and detection of TYR inhibitors in natural products. Besides, more clinical researches are expected to be carried out to develop effective and safe skin whitening agents.

**Table 3.** Summary of the advantages and drawbacks of different separation methods for TYR inhibitors screening.

Separation methods	Advantages	Drawbacks
UF affinity screening	Easy operation, time-saving	Non-specific binding and false-positive results, repetitive manual operation of reverse UF
Immobilised target affinity screening	High stability and durability of the enzymes, low costs, short analysis time	Non-specific binding, possible changes of protein spacial structure, loss of the activities of immobilised enzymes
Offline 2 D-LC/MS affinity screening	Ultra-fast separation of enzyme-ligand complex and small molecules, a low false-positive result	Chromatographic column for separation of large and small molecules needs to be optimised
Online CE-based methods	High-efficiency separation, short analysis time, minimal sample consumption, high sensitivity, easy to realise automatisation	Poor repeatability, only applicable to the activity evaluation of monomers or extracts
(HP)TLC -autography	Rapid and efficient, the capability of simultaneous detection of multiple samples, visualisation of test results	Poor separation effect, low stability of the enzyme, and insufficient sensitivity
HPLC-MS-EIA	Automation, simultaneous acquisition of chemical activity information	High cost, false-negative result of low content compounds, more effort to develop the method
SER	Less time and solvent consumption, low operating costs, and little pollution to the environment	Poor repeatability of fingerprints, no standardised data processing, the requirement of activity verification

## Author contributions

Xiao-wei Zhang, Guang-li Bian, Pei-ying Kang, Xin-jie Cheng and Kai Yan: Writing – original draft. Yong-li Liu and Yan-xia Gao: Investigation and designation of the framework. De-qiang Li: Conceptualisation and writing – review & editing.

## Supplementary Material

Supplemental Material
