# Supplementary material for: Recent advance in the discovery of tyrosinase inhibitors from natural sources via separation methods
Source: J Enzyme Inhib Med Chem. 2021 Sep 27;36(1):2104–17. doi: 10.1080/14756366.2021.1983559 (PMC8480707; doi:10.1080/14756366.2021.1983559)
Supplement: Supplemental Material [file IENZ_A_1983559_SM1326.pdf]

Recent advance in the discovery of tyrosinase inhibitors from natural sources via separation methods

Xiao-wei Zhang[1#], Guang-li Bian[2#], Pei-ying Kang[3#], Xin-jie Cheng[2], Kai Yan[4], Yong-li Liu[4],Yan-xia Gao[4], De-qiang Li[2]*

[1.] Department of Neurological surgery, the Second Hospital of Hebei Medical University, No.215, Heping West Road, Shijiazhuang 050000, Hebei province, People's Republic of China

[2.] Department of Pharmacy, the Second Hospital of Hebei Medical University, No.215, Heping West Road, Shijiazhuang 050000, Hebei province, People's Republic of China

[3.] Department of Clinical Laboratory, the Second Hospital of Hebei Medical University, No.215, Heping West Road, Shijiazhuang 050000, Hebei province, People's Republic of China

[4.] Institute for Drug Control of Hebei Province, No.219, Yuquan Road, Shijiazhuang 050011, Hebei province, People's Republic of China

[#] These authors contributed equally to this work.

* Correspondence to: De-qiang Li, Department of Pharmacy, the Second Hospital of Hebei Medical University, No.215, Heping West Road, Shijiazhuang 050000, Hebei province, People's Republic of China.

E-mail: deqli@163.com (D.Q. Li). Tel: +86 0311 6663630

Table 1S. The chemical structures of identified active compounds

No.	Classification	Compounds	Chemical structure
1		3′-Hydroxypuerarin	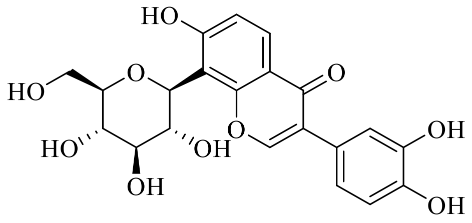
2		Puerarin	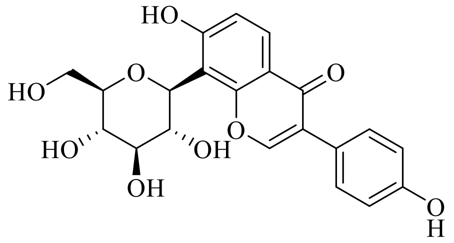
	Flavonoids		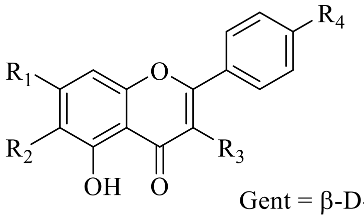 Gent = β-D-gentiobiosyl<br>Glc = β-D-glucopyranosyl<br>GlcA = β-D-glucuronopyranosyl
			R1
3		Oroxin B	O-Gent
4		Kaempferide-3-O-β-D-gentiobioside	OH
5		Chrysin-7-O-β-D-gentiobioside	O-Gent
6		Oroxin A	O-Glc
7		Chrysin-7-O-β-D-glucuronid	O-GlcA
8		Baicalein	OH
9		Chrysin	OH

			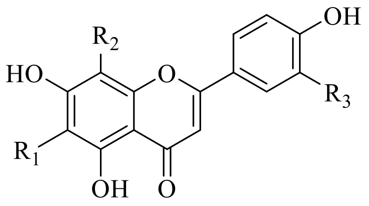<br>Flavonoid		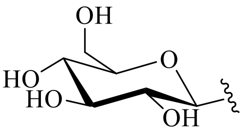<br>β-glucosy1(β-Glc)
			R1	R2	R3
10		Isoorientin	β-Glc	H	OH
11		Vitexin	H	β-Glc	H
12		Isovitexin	β-Glc	H	H
13		Isoorientin 3′-methyl ether	β-Glc	H	OCH_3
14		Quercetin-3-O-(6-O-malonyl)-β-D glucopyranoside	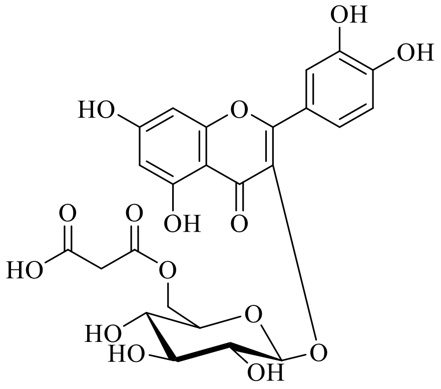<br>Quercetin-3-O-(6-O-malonyl)-β-D-glucopyranoside		
15		Kaempferol-3-O-(6-O-malonyl)-β-D-glucopyranoside	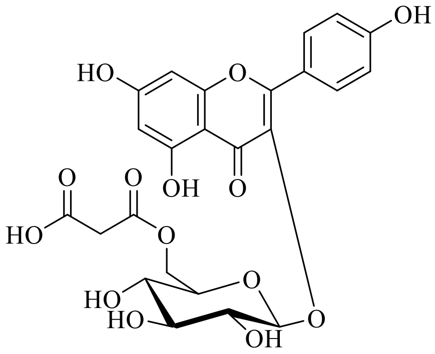<br>Kaempferol-3-O-(6-O-malonyD)-β-D-glucopyranoside		

16		Rutin	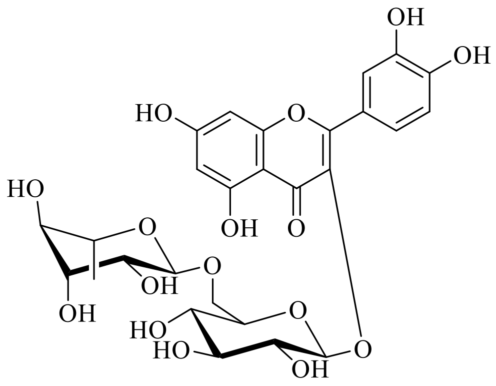<br>Rutin
17		Isoquercitrin	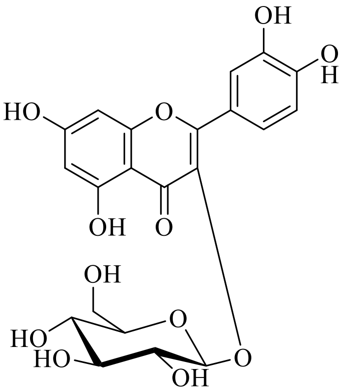<br>Isoquercitrin
18		Astragalin	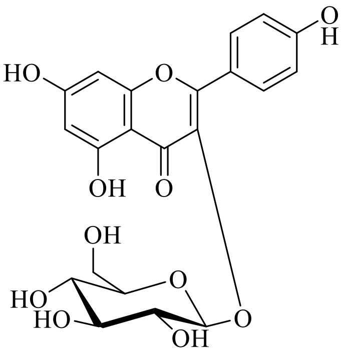<br>Astragalin
19		Kaempferol-3-O-α-L-rhamnopyranosyl-(1-6)-β-D-glucopyranoside	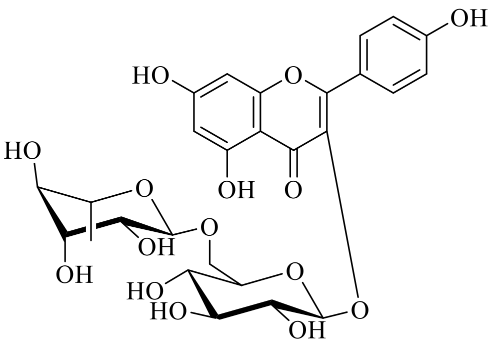<br>Kaempferol-3-O-α-L-rhamnopyranosyl-(1-6)-β-D-glucopyranoside

			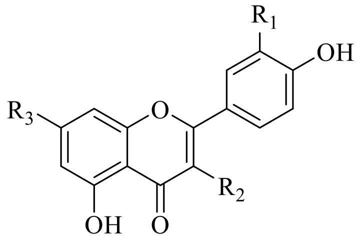 Flavones/flavonols		
			R1	R2	R3
20		Hyperoside	OH	OGal	OH
21		Quercetin-3-O-xyloside	OH	OXyl	OH
22		Luteolin-7-O-glucoside	OH	H	OGlc
23		Quercetin-3-O-arapyranoside	OH	OArapyraniside	OH
24		Quercetin-3-O-arafuranoside	OH	OArafuranoside	OH
25		Kaempferol-3-O-glucoside	H	OGlc	OH
26		Quercetin-3-O-rhamnoside	OH	ORha	OH
27		7-O-Methyl quercetin-3-O-rhamnoside	OH	ORha	OCH_3
28		1, 2, 6-Trihydroxy-5-methoxy-7-(3-methylbut-2-enyl) xanthone	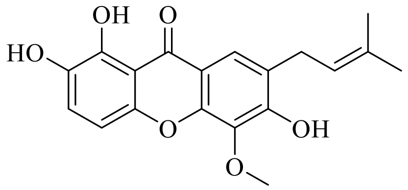 1, 2, 6-Trihydroxy-5-methoxy-7-(3-methylbut-2-enyl)xanthone		
29		7-Hydroxy-2-(2-hydroxy) propyl-5methyl-benzopyran-γ-one	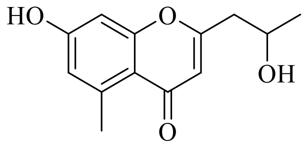 7-Hydroxy-2-(2-hydroxy)propyl-5methyl-benzopyran-γ-one		
30		1, 4, 5, 6-Tetrahydroxy-7,8-bis(3-methylbut-2-enyl) xanthone	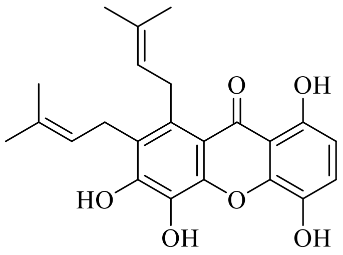 1, 4, 5, 6-Tetrahydroxy-7, 8-bis(3-methylbut-2-enyl) xanthone		

31		Kuwanon G	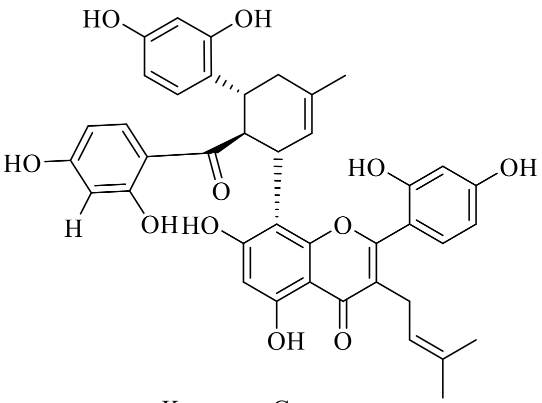 Kuwanon G
32		Kuwanon H	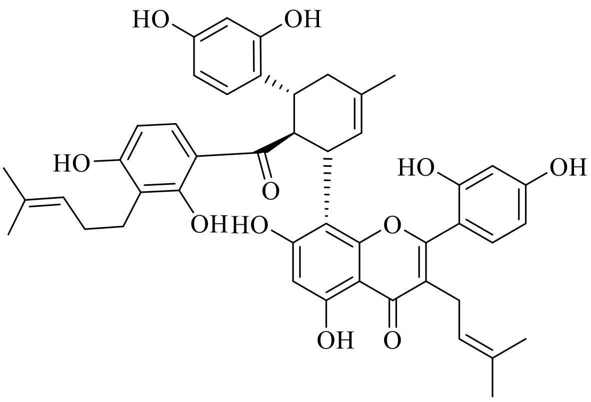 Kuwanon H
33		Puerarin-6''-O-xyloside	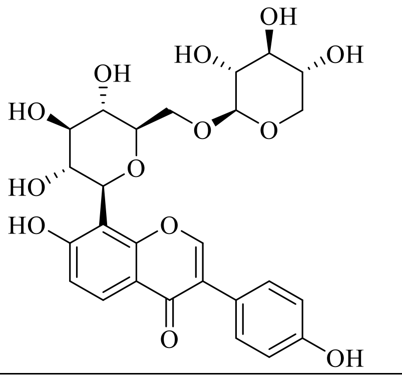
34	Isoflavones	Daidzin	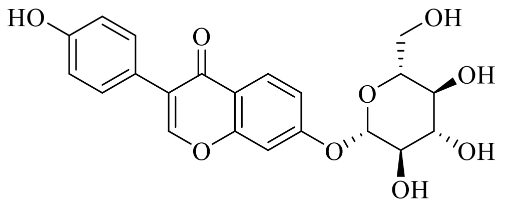
35		Genistin	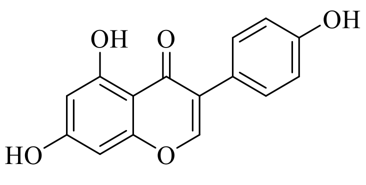

36		6"-O-acetyldaidzin	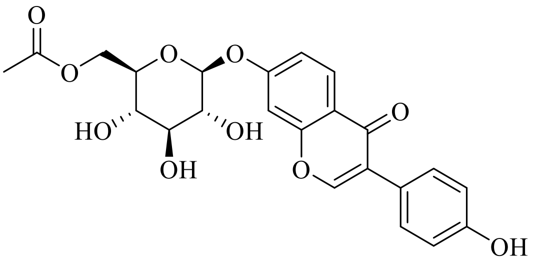		
37		Daidzein	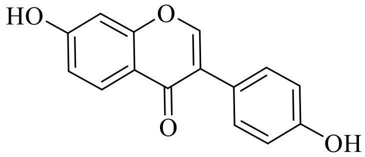		
			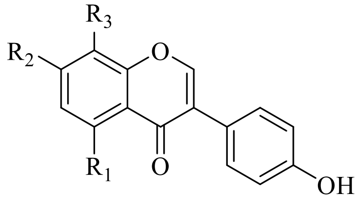		
			R1	R2	R3
38		Mirificin	H	OH	Glc(6,1)-APi
39		Genistinc	OH	OGlc	H
40		Licoarylcoumarin	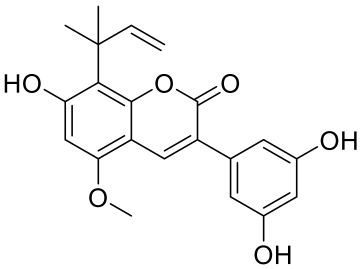		
41		Puerarin-4’-O-glucoside	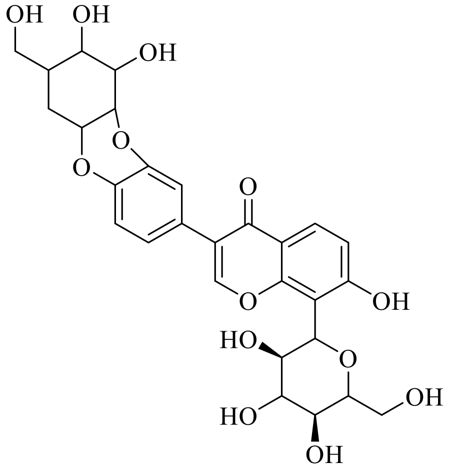 puerarin-4'-*O*-glucoside		

42		3’-Methoxy puerarin	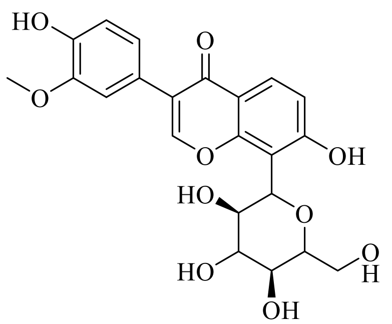 3'-methoxy puerarin	
43		Puerarin apioside	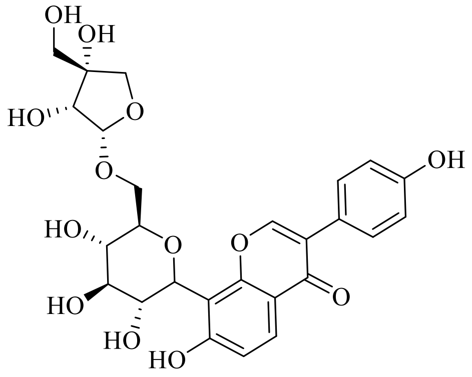 puerarin apioside	
44		Glabridin	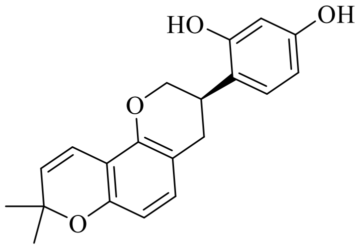	
45		Naringenin	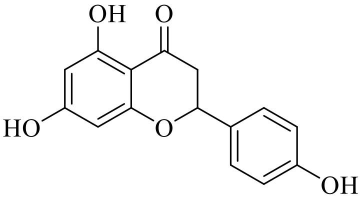	
	Dihydroflavonoids	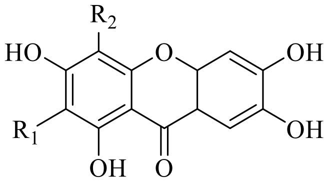 Xanthones	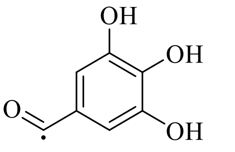 R=Galloyl	
			R1	R2
46		Mangiferin	Glc	H
47		6'-O-Galloyl-mangiferin	6'-O-Galloylglc	H
48		Isomangiferin	H	Glc
49		6'-O-(p-Hydroxybenzoyl) mangiferin	6'-O-ρ-Hydroxybenzoylglc, Rz=H	H

<table>
<tr><td></td><td rowspan="16"></td><td></td><td colspan="3">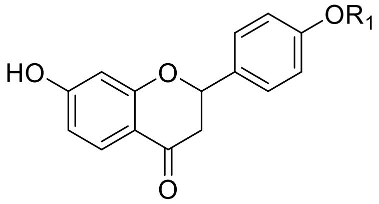</td></tr>
<tr><td></td><td></td><td colspan="3">R1</td></tr>
<tr><td>50</td><td>Liquiritin apioside</td><td colspan="3">APi-Glc</td></tr>
<tr><td>51</td><td>Liquiritigenin</td><td colspan="3">H</td></tr>
<tr><td>52</td><td>Chrysoeriol</td><td colspan="3">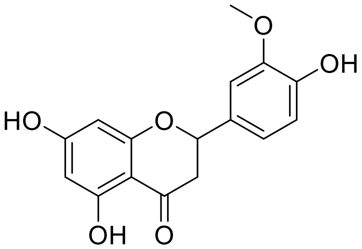</td></tr>
<tr><td></td><td></td><td colspan="3">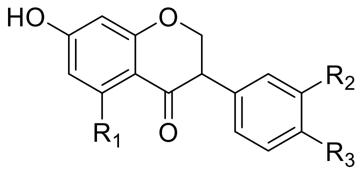</td></tr>
<tr><td></td><td></td><td>R1</td><td>R2</td><td>R3</td></tr>
<tr><td>53</td><td>Dihydrodaidzein</td><td>H</td><td>H</td><td>OH</td></tr>
<tr><td>54</td><td>Glycyrrhisoflavanone</td><td>OCH3</td><td>7-O-(CH3)2CH
=CH-8</td><td>7-O-(CH3)2
CH=CH-8</td></tr>
<tr><td></td><td></td><td colspan="3">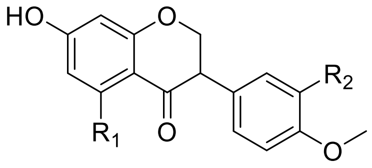</td></tr>
<tr><td></td><td></td><td>R1</td><td colspan="2">R2</td></tr>
<tr><td>55</td><td>Formononetin</td><td>H</td><td colspan="2">H</td></tr>
<tr><td>56</td><td>Pratensein</td><td>OH</td><td colspan="2">OH</td></tr>
<tr><td></td><td rowspan="5">Phenols</td><td></td><td colspan="3">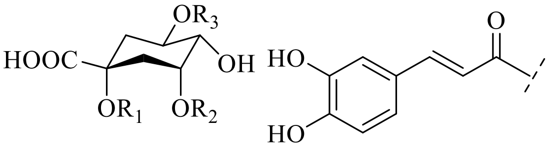
Qunic cid Caffeoyl group</td></tr>
<tr><td></td><td></td><td>R1</td><td>R2</td><td>R3</td></tr>
<tr><td>57</td><td>3,5-Di-O-caffeoylquinic acid</td><td>H</td><td>Caffeoyl</td><td>Caffeoyl</td></tr>
<tr><td>58</td><td>1,5-Di-O-caffeoylquinic acid</td><td>Caffeoyl</td><td>H</td><td>Caffeoyl</td></tr>
<tr><td>59</td><td>Chlorogenic acid</td><td>H</td><td>Caffeoyl</td><td>H</td></tr>
</table>

60		Cryptochlorogenic acid	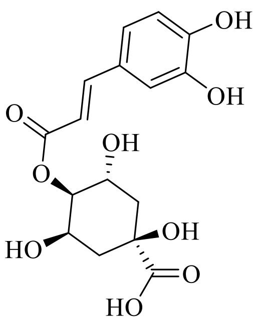<br>Cryptochlorogenic acid
61		3,4-Dicaffeorylquinic acid	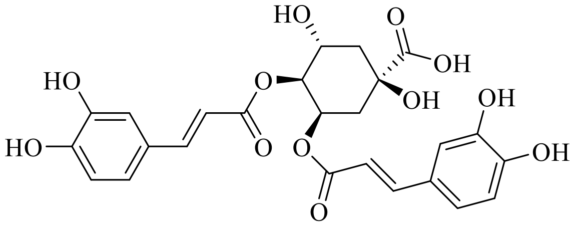<br>3,4-Dicaffeoylquinic acid
62		3,5-Dicaffeorylquinic acid	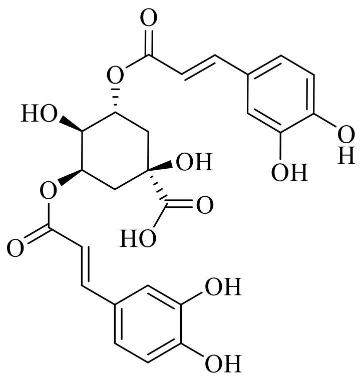<br>3,5-Dicaffeoylquinic acid
63		4,5-Dicaffeorylquinic acid	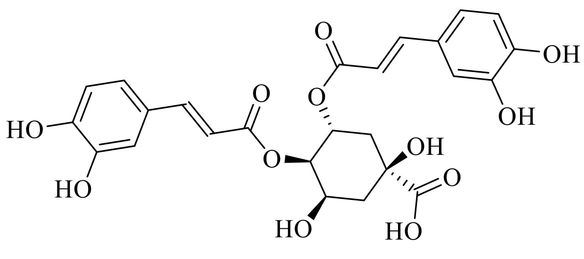<br>4,5-Dicaffeoylquinic acid
64		4,4'-Dihydroxydiphenyl ether	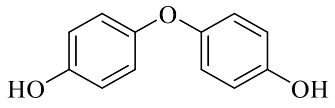

65		4-(1-(4-hydroxyphenyl)phenyl)Phenol	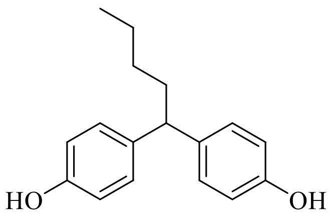				
66		4,4’-Methylenediphenol	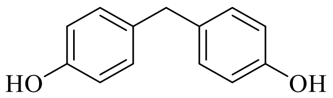				
67		2,4-Bis(4-hydroxybenzyl) phenol	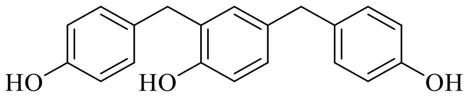				
68		4-Hydroxybenzyl methyl ether	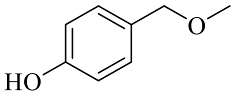				
`			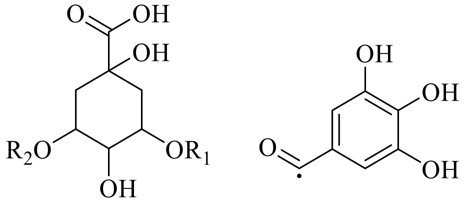 Galloylated quinic acids R=Galloyl				
			R1	R2			
69		3-O-Galloyl quinic acid	Galloyl	H			
70		3,5-Di-O-galloyl quinic acid	Galloyl	Galloyl			
71		5-O-Digalloyl quinic acid	H	5-Digalloyl			
			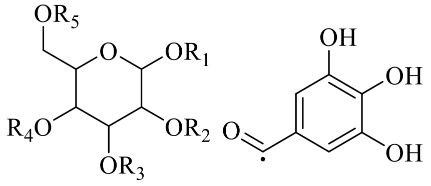 Gallotannins R=Galloyl				
			R1	R2	R3	R4	R5
72		1,4,6-Tri-O-galloyl glucoside	Galloyl	H	H	Galloyl	Galloyl
73		1,3,4,6-Tetra-O-galloyl glucoside	Galloyl	Galloyl	H	Galloyl	Galloyl
74		1,2,3,4,6-Penta-O-galloyl glucoside	Galloyl	Galloyl	Galloyl	Galloyl	Galloyl

75		3-O-Galloyl shikimic acid	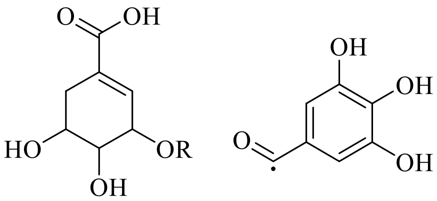 Galloylated shikimic acid R=Galloyl		
76		Epicatechin gallate	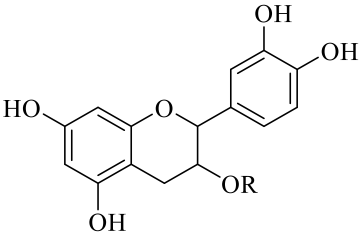 Galloylated catechin		
77		Ethyl 2,4-dihydroxy-3-galloyl Oxybenzoate	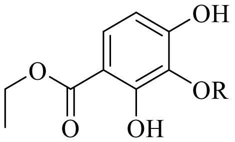		
			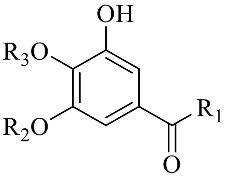		
			R1	R2	R3
78		Gallic acid	H	H	H
79		Ethyl gallate	CH_2CH_3	H	H
80		Digallic acid	H	Galloyl	H
81		Ethyl digallate	CH_2CH_3	H	Galloyl
82		Protocatechuic acid	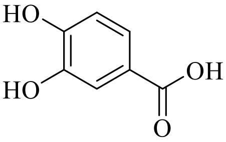		
83		Neolicuroside	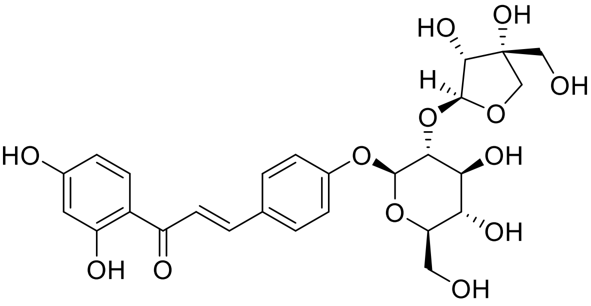		

84		Liquiritin	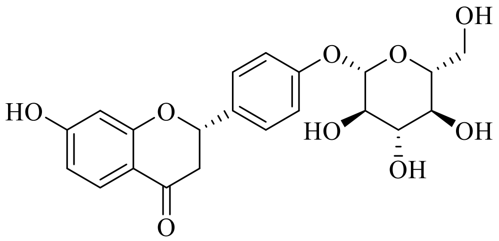Liquiritin
85		Ononin	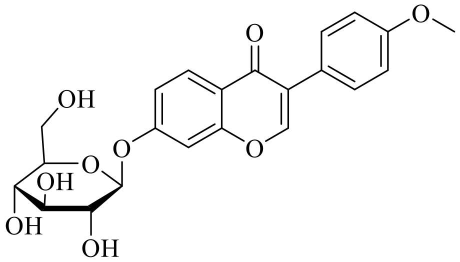
86		3'-Hydroxy puerarin	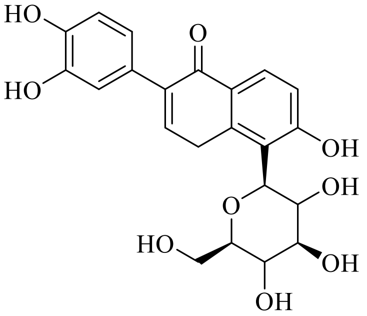3'-hydroxy puerarin
87		Oxyresveratrol	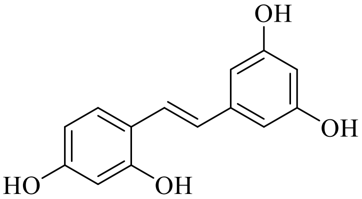
88		Veraphenol-4′-O-β-D-glycoside	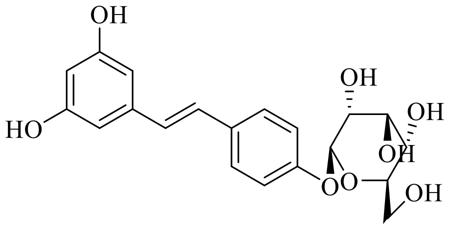Veraphenol-4'-O- β-D- glycoside

89		2-O-cinnamyl-galloyl glucose	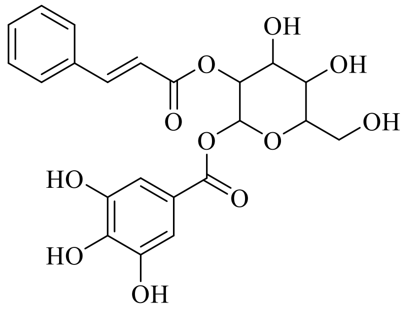 2-O-cinnamyl-galloyl glucose
90		l, 6-Digalloyl-2-cinnamon acyl glucose	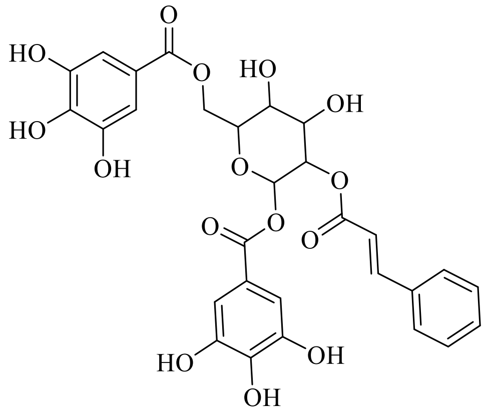 l, 6-Digalloyl-2-cinnamon acyl glucose
91		Protocatechuic aldehyde	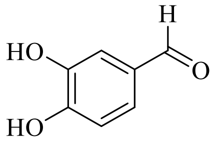 Protocatechuic aldehyde
92		Hydroxysafor yellow A	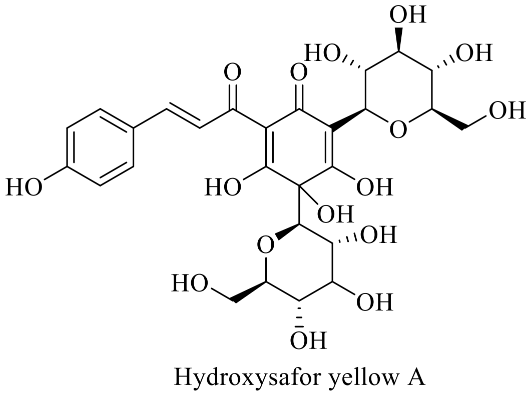 Hydroxysafor yellow A

93		Mulberrofuran G	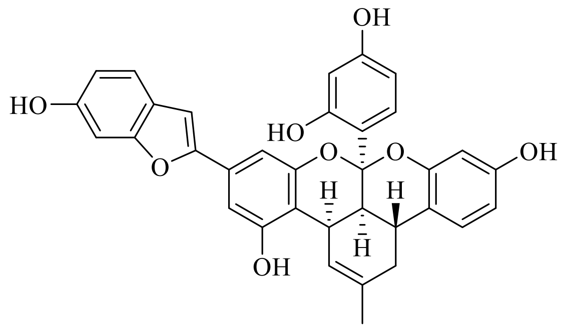 Mulberrofuran G			
94	Organic acid	3-(5-Hydroxybenzofuran -6-yl) propanoic acid	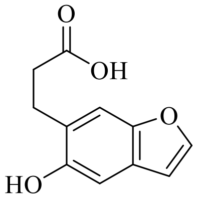 3-(5-hycroxybenzofran-6-yl) propanoic acid			
95	Alkaloids	9-Octadecenamide, (trans-)	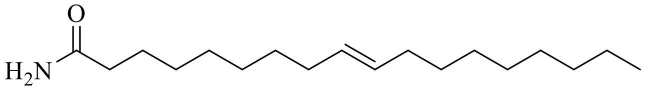			
96		9-Octadecenamide, (cis-)	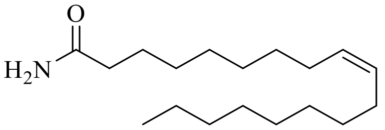			
			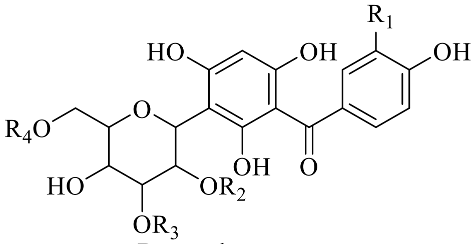 Benzophenones 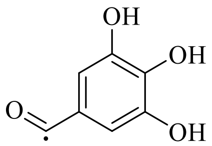 R=Galloyl			
			R1			
97		Iriflophenone 3-C-glucoside	H	R2	R3	R4
98		Iriflophenone 3-C-(2'-O-galloyl)-glucoside	H	H	H	H
99	Glycosides Quinones	Maclurin 3-C-(2'-O-p-hydroxybenzoyl)-glucoside	OH	Galloyl	H	H
100		Iriflophenone 3-C-(2',6'-di-O-galloyl)-glucoside	H	ρ-Hydroxybenzoyl	H	H
101		Iriflophenone 3-C-(2'-O-p-hydroxybenzoyl)-gluco	H	Galloyl	H	Galloyl

		side				
102		Maclurin 3-C-(2'-O-galloyl)-glucoside	OH	ρ-Hydroxybenzoyl	H	H
103		Maclurin 3-C-(2',3'-di-O-galloyl)-glucoside	OH	Galloyl	H	H
104		Iriflophenone 3-C-(2',3',6'-tri-O-galloyl)-glucoside	H	Galloyl	Galloyl	H
			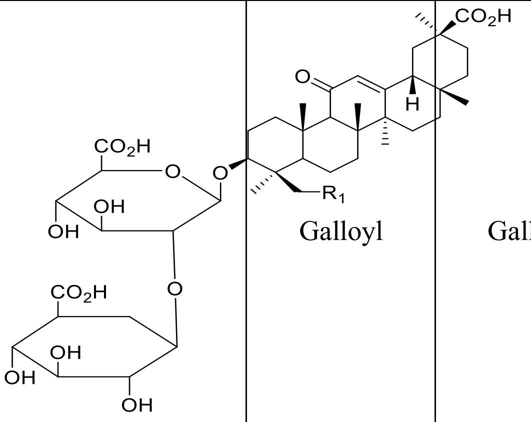	Galloyl	Galloyl	Galloyl
			R1			
105		Licorice saponin G2	OH			
106		Albiforin	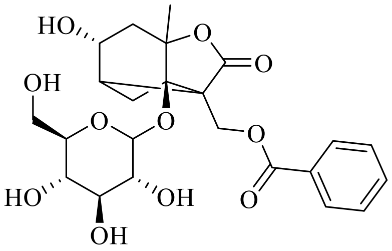 Albiforin			
107		Paeoniforin	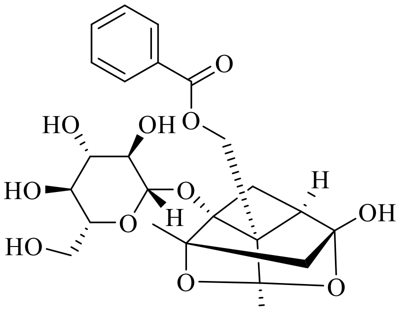 Paeoniforin			

108		Tanshinone IIA	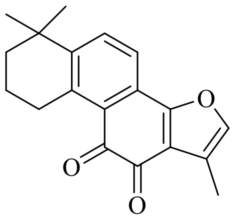<br>Tanshinone IIA
109	Quinones Terpenoids	Emodin methyl ether	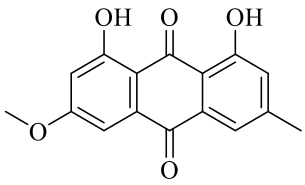<br>Emodin methyl ether
110		ω-Hydroxy aoe-emodin	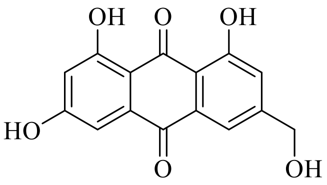<br>ω-Hydroxy aloe-emodin
111		Emodin	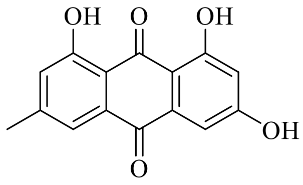<br>Emodin
112		α-Santalol	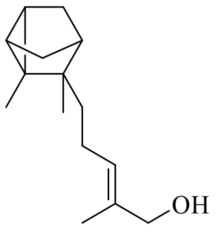
113	Others	1,3-Digalleoyl acetone	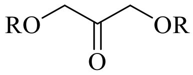
114	Others	Kojic acid	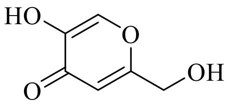
115		Cinnamaldehyde	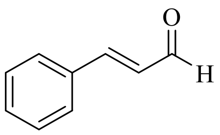

116		5-Hydroxymethyl-furfural	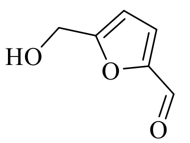
117		Octadecanoic acid, 2,3-Dihydroxypropyl ester	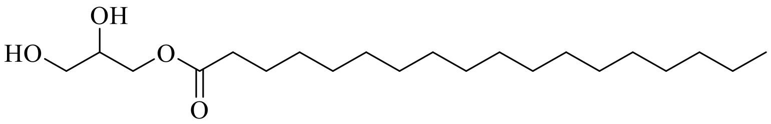
118		4,4'-Dimethoxydiphenyl methane	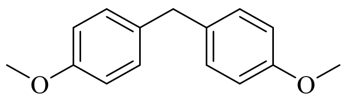
119		12-15 Heneicosadienoic acid, Octadecanoic acid, 2-Hydroxylpropyl esters	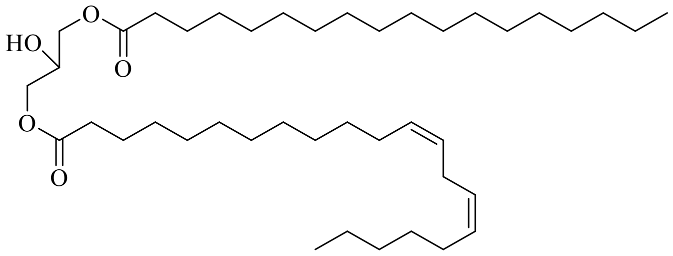
120		3-(4-tert-butylphenyl)-2-Methylpropanal	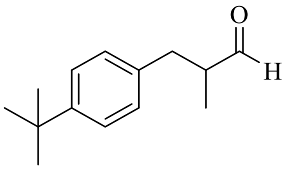
121		4,4'-Dihydroxybenzylsulfid	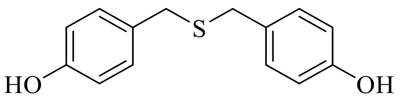
122		Octadecanoic acid, 2-Hydroxy-1-(hydroxymethyl)ethyl ester	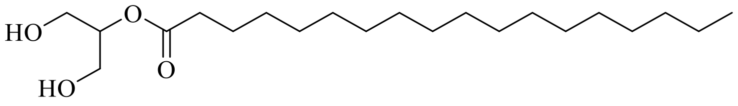
